# The influence of commercially-available carbohydrate and carbohydrate-protein supplements on endurance running performance in recreational athletes during a field trial

**DOI:** 10.1186/1550-2783-10-17

**Published:** 2013-03-28

**Authors:** Adriana Coletta, Dixie L Thompson, Hollie A Raynor

**Affiliations:** 1 Department of Nutrition, The University of Tennessee- Knoxville, 1215 W Cumberland Avenue, 229 Jessie Harris Building, Knoxville, TN, 37996-1920, USA; 2 Department of Kinesiology, Recreation, & Sport Studies, The University of Tennessee- Knoxville, 1914 Andy Holt Avenue, 322 HPER Building, Knoxville, TN, 37996-2700, USA

**Keywords:** Field experiment, Endurance running, Sport drinks, Carbohydrate-protein

## Abstract

**Background:**

It is recommended that endurance athletes consume carbohydrate (CHO) supplements, providing 6-8% CHO concentration, during exercise > 60 minutes to improve athletic performance. Recently research has compared carbohydrate-protein (CHO-P) supplementation to the traditionally used CHO supplementation during endurance exercise, following these supplementation recommendations, in controlled settings, but not under simulated applied conditions such as a field trial. Therefore, the purpose of the present investigation was to test CHO and CHO-P supplementation under applied conditions such that commercially-available isocaloric (CHO-P & double-carbohydrate [CHO-CHO]) and isocarbohydrate (CHO-P & CHO) supplements were compared to a placebo (PLA), within an outdoor running field trial > 60 minutes in order to asses their influence on endurance performance.

**Methods:**

Twelve male recreational runners completed four, 19.2 km runs, where they were instructed to run at a pace similar to race pace including a final sprint to the finish, which in this case was the final two laps of the course (1.92 km). Supplementation was provided before the start and in 4 km increments. Performance was measured by time to complete the 19.2 km run and last 1.92 km sprint.

**Results:**

Analyses found no difference between supplements in time to complete the 19.2 km run (PLA = 88.6 ± 11.6 min, CHO = 89.1 ± 11.3 min, CHO-P = 89.1 ± 11.8 min, CHO-CHO = 89.6 ± 11.9 min) or last 1.92 km sprint to the finish (PLA = 8.3 ± 1.2 min, CHO = 8.2 ± 1.2 min, CHO-P = 8.2 ± 1.2 min, CHO-CHO = 8.4 ± 1.5 min).

**Conclusions:**

When following recommendation for supplementation within a field trial, commercially available CHO and CHO-P supplements do not appear to enhance performance in male recreational runners.

## Background

Guidelines for nutrient timing and amounts for endurance exercise are well known to endurance athletes competing at the recreational and elite levels. Caloric supplementation, providing 6-8% carbohydrate (CHO) concentration or 30–60 grams of CHO per hour, is recommended during exercise lasting > 60 minutes at moderate- to vigorous-intensity to enhance athletic performance [1-4]. Post-exercise, consumption of carbohydrates and protein, ideally within a 3:1 CHO to protein ratio, is warranted to replenish muscle glycogen and enhance muscle recovery [2].

More recently, the use of carbohydrate-protein (CHO-P) supplementation during endurance exercise has been rigorously tested within controlled laboratory settings using cycling ergometer protocols to examine if CHO-P supplementation enhances performance over that of the traditionally used CHO supplement [5-14]. Mixed results have been found, which may be a consequence of variances in study design and methodology.

CHO and CHO-P supplements, such as Gatorade® (Gatorade, Inc., Chicago, IL) and Accelerade® (PacificHealth Laboratories, Inc; Woodbridge, NJ) respectively, are commonly available to recreational athletes and are marketed with the premise of enhancing athletic performance. Thus, it is important to compare commercially-available supplements within trials more closely representing applied field use, as opposed to controlled laboratory settings in recreational athletes to evaluate their ability to enhance performance. Two studies have compared commercially-available CHO supplements to PLA in competitive runners within a field experiment [15,16]. Both studies found no significant difference in endurance running performance between CHO supplementation and PLA [15,16]. Only one investigation has compared commercially-available CHO and CHO-P supplements to a PLA on endurance performance in competitive cyclists and found no differences in performance when comparing CHO, CHO-P, and PLA [17]. However, this investigation was conducted within a controlled laboratory setting using a cycling ergometer protocol [17]. To date, no investigation has tested commercially-available CHO and CHO-P supplements within a field experiment in recreational athletes. Therefore, the purpose of the present investigation was to assess the influence of commercially-available CHO and CHO-P supplements on endurance performance, while simulating real-life endurance running conditions in recreational athletes.

## Methods

### Study design

This study used a randomized, latin-square (4 × 4), crossover, placebo-controlled design [Table 1]. Order of supplementation was the between-subject factor and type of supplementation (PLA, CHO, CHO-CHO, and CHO-P) was the within-subject factor. The primary dependent variables were the time to complete the last 1.92 km sprint to the finish and the 19.2 km run. The study was registered at ClinicalTrials (NCT00972387), a registry of clinical studies conducted in the U.S.

**Table 1:** 4 x 4 Latin square design

	**Trial order 1**	**Trial order 2**	**Trial order 3**	**Trial order 4**
Time Trial 1	CHO	CHO-P	CHO-CHO	PLA
Time Trial 2	CHO-P	CHO-CHO	PLA	CHO
Time Trial 3	CHO-CHO	PLA	CHO	CHO-P
Time Trial 4	PLA	CHO	CHO-P	CHO-CHO

*Note. CHO = Carbohydrate; CHO-P = Carbohydrate-Protein; CHO-CHO = Double Carbohydrate; PLA = Placebo.

### Participants

Twelve male recreational runners were recruited from both the University of Tennessee campus and a local running club. Eligibility criteria included: males; 18–55 years old; engaged in runs 45-90+ minutes ≥ 4 days/week for the previous 4 weeks and ≥ 16 km for 2–4 occasions/month; body mass index (BMI) 18.50-24.99 kg/m^2^; no allergies to products containing soy, milk, or aspartame; and no history of heart conditions, and/or recently experienced shortness of breath, chest pain, bone, or joint problems during running or daily activities. Thirty-eight individuals expressed interest in participating and were phone-screened for eligibility. Of the 38 individuals screened, 21 did not meet eligibility: 7 had a time commitment conflict, 6 had reported BMI ≥ 25 kg/m^2^, 5 were no longer interested in participating after learning more about the study, 2 had transportation conflicts, and 1 had a food allergy. Three participants were eligible, but did not participate as they were unable to be contacted following the screening. Thus, from the 38 individuals that were phone-screened, informed consent was collected from 17 participants. Of these 17 consented participants, 5 withdrew from the study: 3 had a time conflict and 2 experienced athletic injuries unrelated to the study. Twelve participants completed the study.

Prior to taking part in the study, participants signed an informed consent form, approved by the University of Tennessee- Knoxville Institutional Review Board.

### Sample size

Sample size calculations presumed 2-sided hypothesis testing, with type one error rate (alpha) = 0.05. Calculations were based on effect sizes reported in the only investigation to date to compare isocaloric and isocarbohydrate supplements to a PLA [13]. To reject with 80% power the null hypothesis versus the alternative that supplement difference is d ≥ 3.90 (cohen’s d effect size) exhibiting greater endurance performance for caloric supplements versus PLA, 8 males were needed [13]. To reject with 80% power the null hypothesis versus the alternative that the supplement difference is d ≥ 1.84 (cohen’s d effect size) exhibiting greater endurance performance for isocaloric supplements versus CHO, 12 males were needed [13].

### Supplements

The supplements used in the present investigation were commercially available in order to increase the external validity of the findings. The PLA used was Crystal Light® (Kraft Food, Inc.). The use of an artificially sweetened placebo is consistent with previous placebo-controlled research [6,7,13,14]. The CHO supplement was Gatorade® (Gatorade, Inc., Chicago, IL), and the CHO-P supplement was Accelerade® (PacificHealth Laboratories, Inc; Woodbridge, NJ). Both the CHO and CHO-P supplements were matched in carbohydrate content (isocarbohydrate) and so a third caloric supplement, double carbohydrate (CHO-CHO) supplement, was tested in order to match the CHO-P supplement in calories (isocaloric). The CHO-CHO supplement was made from Gatorade® (Gatorade, Inc., Chicago, IL). The purpose of testing isocaloric and isocarbohydrate supplements was to observe if any previously examined performance benefits from CHO-P supplementation was attributed to the presence of protein or additional calories per serving in the CHO-P supplement compared to the traditionally used CHO supplement.

All supplements were in the powder form and were mixed with water in a blender to ensure uniform viscosity and mouth-feel. Supplements also consisted of the same color and flavor (fruit punch). All drinks were made following manufacturer instructions. Both the CHO and CHO-P supplements were matched with 6% CHO concentration but varied in total calories per serving, 25 kcal vs. 40 kcal respectively. The CHO-P supplement also included 1.4% protein concentration. The CHO-CHO supplement matched the CHO-P supplement in total calories per serving, 40 kcal, and consisted of 9% CHO concentration.

### Procedures & measures

Before the initial session, participants were emailed standard pre-test protocols to follow for body composition and VO_2_max tests to ensure measurements were accurate. At the start of the initial session, informed consent, approved by The University of Tennessee Institutional Review Board, was reviewed and signed. Height was recorded to the nearest centimeter using a stadiometer (Sunbeam Products Inc, Boca Raton, FL). Weight was recorded to the nearest 0.2 kg using the electronic scale associated with the BOD POD (COSMED USA Inc., Concord, CA). Body composition was measured via whole body air-displacement plethysmography technique with the BOD POD. Participants were dressed in standard BOD POD protocol attire while measurement was conducted.

Next, participants completed a treadmill VO_2_max test. Running speed was self-selected and remained constant throughout the test. The test began at 0% grade and increased 1% in one-minute increments until the participant reached volitional exhaustion. Body composition and VO_2_max tests were conducted to describe participant characteristics.

Following the treadmill test, the first run was scheduled no more than one week after the initial session and participants were provided a 24-hour diet/exercise record to record all caloric food/beverage intake and aerobic exercise during the 24 hours before the run. Participants were instructed to keep diet and aerobic exercise consistent before all runs in order to minimize variances in glycogen status and physical condition among trials. All trials were scheduled 7–10 days apart.

At each run, participants submitted the diet/exercise record. Based upon the initial diet record presented at the first trial, participants received a diet prescription for the 24 hours before the remaining trials (derived from the quantified serving sizes in the Diabetes Exchange System) and a copy of their original food record as an example. To compare the previous 24-hour dietary intake and the last meal consumed prior to each run between the sessions, total calories and percent calories from each macronutrient were analyzed using the NDS-R computer diet analysis program version 2008 (NCC, University if Minnesota, Minneapolis, MN). Glycogen status was estimated based on guidelines stating 8–12 hour time period without consuming calories results in significant depletion of glycogen stores [18]. Since all participants consumed calories within eight hours before any run, no participant was considered glycogen-depleted. In addition, an aerobic exercise prescription for the 24 hours before the remaining trials was provided. This prescription was based upon the initial aerobic exercise record presented at the first trial, and participants were given a prescription of +/− 30-minute variance from the amount of aerobic exercise conducted in the 24 hours prior to the first run. No trials were re-scheduled due to participant noncompliance with exercise prescription.

Before each run, diet/exercise records were reviewed and weather conditions measured on site (temperature, humidity level, average wind speed [Ambient Weather, Chandler, AZ]). All running trials were conducted on a somewhat isolated, outdoor, paved, closed running trail surrounding a lake, with one lap = 0.96 km. As in Burke and colleagues investigation (2005), the course location was selected to help with controlling wind and other weather conditions [15]. For each trial, participants were instructed to run with intensity similar to race pace, providing an all-out sprint for the last two laps, 1.92 km, in order to simulate the final kick typically used within training and competition. Exercise intensity was assessed using Borg 10-point scale of perceived exertion (RPE) [19] at the mid-point and finish, and heart rate (HR) at the start of the run, start of the last two laps, and finish via downloadable Polar s625x HR monitor (Polar Electro Inc., Lake Success, NY). Total time was measured via Timex IronMan® stopwatch (TIMEX Group USA Inc., Middlebury, CT); at the start of the last two laps, time elapsed was recorded and the difference between this start-time and finish-time of the run was calculated to determine time for 1.92 km.

Supplementation was administered in 120 ml servings 5 minutes before the start, and every 4 km throughout the run (600 ml total). Supplements were provided in 177 ml plastic cups. Before the start of a run, participants consumed the entire contents of a cup in front of the investigator. Supplementation during the run emulated water stations used in marathons. Participants were instructed to consume the entire contents of the cup within a marked distance of 160 meters from drinking station. This distance was in view of the investigator so consumption of the supplement could be verified. Supplementation was not administered at the finish; however, participants were allowed water ad libitum.

### Statistical analyses

Baseline characteristics were analyzed using one-way analysis of variance (ANOVA), with supplementation order group as the between-subject factor. Mixed-factor ANOVAs were used for analyses of diet (energy and percent energy from macronutrients consumed in the previous 24-hours and in the meal prior to each run), minutes since last eaten prior to the trial, exercise, and weather conditions, with supplementation order as between-subject factor and supplement type (PLA, CHO, CHO-CHO, and CHO-P) as within-subject factor. For analyses of exercise intensity, mixed-factor ANOVAs were used, with a between-subject variable of supplementation order and within-subject variables of supplement type and time (RPE: mid-point, finish; HR: start, start of last two laps, finish).

A significant difference was found between supplement-type for percent calories from protein (% kcals PRO) consumed in the previous 24 hours. To control for this difference, a regression analysis was conducted on the primary dependent variable, time to complete the 19.2 km run, using % kcals PRO as the independent variable. Additionally, for the analyses on the last 1.92 km, along with controlling for % kcals PRO, time to complete the previous portion of the 19.2 km was controlled, thus a regression analysis was conducted on the primary dependent variable of time to complete the last 1.92 km, using % kcals PRO and time to complete the previous portion of the 19.2 km as the independent variables. Four sets of residualized values, one for each supplement type, were used in mixed-factor ANOVAs, with supplementation order used as the between-subject variable and supplement type as the within-subject variables, to analyze time to complete the 19.2 km run and the last 1.92 km. Probability levels were based on the Greenhouse-Geisser test to control for sphericity in the mixed-factor ANOVAs. Post hoc comparisons with Bonferroni corrections were used for significant outcomes. Data were analyzed using SPSS statistical software, version 18.0 (Chicago, IL), with alpha set *a priori* at *P* < 0.05. For each caloric supplement during the 19.2 km time trial (TT) and final 1.92 km of the course, effect size was reported, Cohen’s *d*, and calculated using G Power [20].

## Results

No significant differences existed between supplementation order in participant demographics, anthropometrics, and VO_2_max values [Table 2]. All participants were Caucasian, aged 32.4 ± 9.5 years, had a BMI of 22.7 ± 1.5 kg/m^2^, and average body composition of 11.2 ± 5.8% body fat. VO_2_max averaged 59.7 ± 7.5 mL/kg/min.

**Table 2:** **Demographic, anthropometric and VO**_**2**_**max measurements (M ± SD)**

	**Trial order 1**	**Trial order 2**	**Trial order 3**	**Trial order 4**
	**n = 3**	**n = 3**	**n = 3**	**n = 3**
Age (years) *p = 0.123*	26.6 ± 4.0	26.6 ± 1.1	34.0 ± 14.0	42.3 ± 6.4
Height (cm) *p = 0.184*	172.2 ±4.3	179.3 ± 8.4	168.4 ± 8.9	179.3 ± 0.5
Weight (kg) *p = 0.173*	66.8 ± 2.8	70.5 ± 13.0	62.4 ± 7.8	77.9 ± 1.1
BMI (kg/m^2^) *p = 0.289*	22.7 ± 1.8	21.9 ± 2.2	22.0 ± 0.8	24.2 ± 0.2
%FFM *p = 0.693*	89.7 ± 7.6	90.8 ± 3.1	89.4 ± 0.8	85.0 ±9.4
%BF *p = 0.706*	10.3 ± 7.6	9.2 ± 3.1	10.6 ± 0.8	14.9 ± 9.4
VO_2_max (mL/kg/min) *p = 0.673*	62.0 ± 7.3	61.0 ± 6.1	61.1 ± 10.7	54.6 ± 7.3

*Note. kg/m2 = Kilograms per Meters Squared; %FFM = Percent Fat Free Mass; %BF = Percent Body Fat; mL/kg/min = Millileters per Kilogram per Minute.

No significant differences were found for total energy (2372 ± 739 kcals) and % kcals CHO (50.2 ± 13.5%) or % kcals fat (31.2 ± 14.2%) consumed in the 24 hours prior to each supplement session. No significant differences were found for total energy (366 ± 226 kcals) and % kcals CHO (57.9 ± 21.2%), % kcals fat (27.1 ± 16.0%), and % kcals PRO (15.0 ± 8.5%) in the meal consumed in the 24 hours prior to each supplement session. A significant main effect of supplement was found for % kcals PRO (F(3,24) = 4.08, p < 0.05), such that % kcals PRO consumed before the CHO-CHO supplement was significantly greater than % kcals PRO consumed before the CHO-P supplement (19.2 ± 9.3% vs. 16.1 ± 12.5%, p = 0.042). No significant difference was found for minutes since last meal consumed prior to each supplemental session (133.8 ± 133.4 minutes). No significant differences were found for amount of aerobic exercise occurring in the 24 hours prior to each supplemental session (53.3 ± 67.9 min). Environmental factors (temperature [11.9 ± 7.2 °C]; percent relative humidity [57.8 ± 12.5%]; and wind speed [0.6 ± 0.5 mph]) were not significantly different between supplemental sessions.

**Figure 1:**
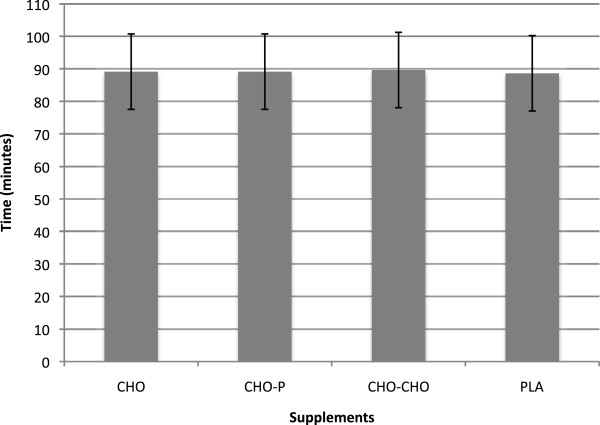
**Time to complete 19.2 km time trials for each supplement (M ± SD).** N = 12; CHO = Carbohydrate; CHO-P = Carbohydrate-Protein; CHO-CHO = Double Carbohydrate; PLA = Placebo.

**Figure 2:**
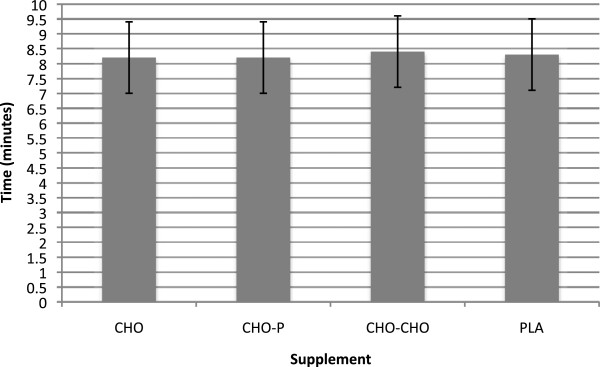
**Time to complete final 1.92 km sprint to the finish (M ± SD).** N = 12; CHO = Carbohydrate; CHO-P = Carbohydrate-Protein; CHO-CHO = Double Carbohydrate; PLA = Placebo.

A main effect of time was found for RPE and HR (p < 0.001). RPE and HR increased at all points measured (4.7 ± 0.7; 9.7 ± 0.9, F(1,24) = 395.49; 84.4 ± 14.5 bpm, 166.0 ± 8.3 bpm, 178.8 ± 7.4 bpm, F(2,48) = 581.08). No significant differences in time to complete the run (PLA = 88.6 ± 11.6 min; CHO = 89.1 ± 11.3 min; CHO-P = 89.1 ±11.8 min; CHO-CHO = 89.6 ± 11.9 min) (Figure 1), or sprint to finish (PLA = 8.3 ± 1.2 min; CHO = 8.2 ±1.2 min; CHO-P = 8.2 ± 1.0 min; CHO-CHO = 8.4 ± 1.5 min) (Figure 2) was found among supplemental session. The effect size between any of the supplements and PLA on endurance performance was very small (d = 0.1). Effect size between PLA and CHO, CHO-P, and CHO-CHO supplements during the 19.2 km run, favoring PLA, was 0.06, 0.059, 0.1, respectively. In addition, for the last 1.92 km of the course, the effect size between PLA and CHO and PLA and CHO-P, favoring the caloric supplements, was 0.08; effect size between PLA and CHO-CHO, favoring the PLA, was 0.07.

## Discussion

The use of CHO-P supplementation during exercise, as opposed to CHO supplementation, is a rising trend among endurance athletes. Previous research has found mixed outcomes regarding CHO-P supplementation and endurance performance enhancement, with all investigations conducted within controlled laboratory settings [5-14]. No investigations testing CHO-P supplementation has been conducted within a field trial under applied, real-life conditions; therefore, the purpose of the present investigation was to assess the influence of commercially-available CHO and CHO-P supplements on endurance running performance within a field setting in order to elicit more practical outcomes for the recreational athlete. Outcomes indicated that there was no difference in athletic performance between commercially-available CHO and CHO-P supplementation during an endurance run while following recommendations for supplementation. This investigation also found that caloric supplementation did not enhance performance above that of the artificially sweetened PLA.

Considering the nature and conditions of the present investigation, it is important to note the strengths in relation to external validity. In this investigation, supplements were compared within trials using an outdoor course that more closely approximated real-life competitive conditions. Additionally, commercially-available supplements were tested, and supplement volume and administration protocol mimicked refueling stations during road races. A glycogen-depleting protocol was not used prior to testing any of the supplements since this is not typical practice of an endurance runner prior to training and competition.

The few running field experiments testing commercially-available CHO supplements against PLA, have also found no effect of supplementation on endurance performance [15,16]. Similar to the present investigation, both investigations conducted trials on an outdoor paved running trail using similar distances for the running trials (18 km [16] vs 20.9 km [15] vs 19.2 km in the present investigation) which resulted in an exercise bout > 60 minutes, controlled for weather conditions and dietary factors, excluded use of a glycogen-depleting protocol prior to supplement testing, provided commercially available supplements in a similar serving size (150 ml vs 120 ml in the present investiation), and administered supplements mimicking real-life conditions (i.e.- water stations as used in a marathon). Based on similarities in methodology and findings among previous running field trials and the present investigation, one may infer that caloric supplementaiton during endurance running may not enhance endurance performance over that of a PLA during runs around 18–20 km in length.

Furthermore, there are two methods commonly used when assessing endurance performance, time trial (TT) and time to exhaustion (TTE). The methodology used in the present investigation and aforementioned field experiments [15,16] most closely resembles TT. Within the TT method, participants exercise for a set period of time or distance. Within TTE, participants are instructed to either cycle at a consistent intensity level, ≥ 65% VO_2_max, until complete fatigue, or cycle at varying intensity levels and at the final level continue until fatigue. When comparing methodologies, the TT method has shown to be more reliable in comparison to TTE such that the calculated coefficient of variance for TTE among several studies has shown to range from 5.2-55.9% whereas as the TT method has demonstrated a variation of 1-5% [17]. Thus, it has been suggested that the TT method may be the best method for assessing endurance performance since it is the most reliable and reproducible of the two methods [17]. Within the current investigation, given the close resemblance to the TT method and high external validity one may infer that results may be directly translated to use for the recreational endurance runner.

Along with this, supplements tested in the present investigation followed current guidelines for CHO supplementation during endurance exercise; thus one would have expected to exhibit a difference in athletic performance between all caloric supplementation and PLA. The previously aforementioned running field trials also followed current guidelines for CHO supplementation. It is important to note the current recommendation for CHO supplementation is based on experiments conducted in controlled laboratory settings comparing CHO supplementation to water using cycling ergometer protocols [21]. Therefore, findings from the present investigation and previous running field studies provide evidence to suggest that investigations conducted within a laboratory setting using a cycling ergometer protocol may not translate directly into field use and generalize to all modes of exercise.

Limitations of the present investigation include a fairly homogenous sample, self-reported diet and exercise prior to each session, and slightly different sources of CHO in the CHO-P vs. CHO and CHO-CHO supplements. To clarify outcomes, future research should compare CHO and CHO-P supplements to PLA in recreational athletes, within field settings, with varying modes of exercise (i.e.- cycling and running), using differing lengths of performance.

## Conclusions

Overall, results of the present investigation suggests no difference in endurance performance between commercially-available CHO and CHO-P supplements in outdoor runs > 60 minutes at moderate- to vigorous-intensity for male recreational runners. Additionally, this supplementation did not enhance performance above that of PLA. As suggested by Burke and colleagues [15], improvements in endurance performance > 60 minutes with CHO supplementation, or any caloric supplementation, warrants further investigation, particularly in regards to translating outcomes to applied use.

## Abbreviations

CHO: Carbohydrate; CHO-P: Carbohydrate-protein; CHO-CHO: Double carbohydrate; PLA: Placebo; BMI: Body mass index; % kcals: Percent calories; RPE: Rating of perceived exertion; HR: Heart rate; ANOVA: Analysis of variance; TT: Time trial; TTE: Time to exhaustion

## Competing interests

The authors declare that they have no competing interests.

## Authors’ contributions

AC conceived the study. AC and HR developed the design of the study. AC recruited participants, screened participants, collected all data, developed all sport drinks tested, performed statistical analyses, and wrote the manuscript. HR helped to draft the manuscript. DL contributed to the study design and helped draft the manuscript. All authors read and approved the final manuscript.

## Authors’ information

This investigation was the master’s thesis research of AC. HR was AC’s thesis advisor and mentor. DL was a member of the thesis committee.
